# Individual differences in exploration and persistence: Grit and beliefs about ability and reward

**DOI:** 10.1371/journal.pone.0203131

**Published:** 2018-09-04

**Authors:** Gillian Dale, Danielle Sampers, Stephanie Loo, C. Shawn Green

**Affiliations:** Department of Psychology, University of Wisconsin-Madison, Madison, WI, United States of America; Kyoto University, JAPAN

## Abstract

The tradeoff between knowing when to seek greater rewards (exploration), and knowing when to settle (exploitation), is critical to success. One dispositional factor that may modulate this tradeoff is “grit.” Gritty individuals tend to persist in the face of difficulty and consequently experience greater life success. It is possible that they may also experience a greater tendency to explore in a reward task. However, although most exploration/exploitation tasks manipulate beliefs about the presence/magnitude of rewards in the environment, the belief of one’s ability to actually achieve a reward is also critical. As such, we investigated whether individuals higher in grit were more likely to explore, and how beliefs about the magnitude/presence of rewards, and the perceived ability to achieve a reward, modulated their exploration tendencies. Over two experiments, participants completed 4 different exploration/persistence tasks: two that tapped into participant beliefs about the presence/magnitude of rewards, and two that tapped into participant beliefs about their ability to achieve a reward. Participants also completed measures of dispositional grit (Experiment 1a and 1b), conscientiousness (Experiment 1b), and working memory (Experiment 1a and 1b). In both experiments, we found a relationship between the two “belief of rewards” tasks, as well as between the two “belief of ability” tasks, but performance was unrelated across the two types of task. We also found that dispositional grit was strongly associated with greater exploration, but only on the “belief of ability” tasks. Finally, in Experiment 1b we showed that conscientiousness better predicted exploration on the “belief of ability” tasks than grit, suggesting that it is not grittiness per se that is associated with exploration. Overall, our findings showed that individuals high in grit/conscientiousness are more likely to explore, but only when there is a known reward available that they believe they have the ability to achieve.

## Introduction

We are often presented with situations in which we have to choose between settling for what we currently have, or pushing forward into an uncertain future to find something better. Should I take the orange that is currently in my hand, or should I instead continue to look through the display at the supermarket for a better orange? Should I order the dish that I already know that I like, or should I try a new dish at the restaurant that might be better? Explore too little and you risk missing the best option; explore too long and you risk wasting time on worse options. This tension between continuing to explore versus settling on the current best option is known in the reinforcement learning literature as the exploration-exploitation tradeoff [1–4]. Due to its pervasiveness across different domains and contexts, it is one of the more well-studied problems in the field. Indeed, there are a great many proposed resolutions to this problem ranging from simple heuristics to highly complex calculations of all possible future outcomes [2,3,5–7].

With respect to human behavior, research has identified several factors that influence the extent to which exploration/exploitation behaviors are shown. These factors include uncertainty over the possibility of reward [5,8], familiarity with the task or environment [9], or the relative cost-benefit tradeoffs of continuing to explore [1,3,5,9], all of which contribute to the exploration/exploitation behaviors (see [9] for a review). These behaviors have been mapped to a variety of neural characteristics (see [10,11] for reviews), and are particularly related to the prefrontal and striatal dopaminergic system [12–14], and the locus coeruleus–norepinephrine system [15,16].

Interestingly, the types of inferences that are required in exploration-exploitation-type situations in the reinforcement learning literature are typically based solely on the probability and/or magnitude of rewards in the environment. In these cases, obtaining a certain reward is a simple matter of exploring long enough to find it. There are some tasks that humans face that have a similar feel, such as in the example given above which suggests that, given enough time, one can explore all of the oranges in the display and thus be guaranteed to find the best option. For many real-world tasks, however, human beings must make an additional inference beyond whether there is a better alternative. Namely, we must assess the probability that we ourselves are actually *capable of* obtaining that better alternative. For instance, when determining whether to take an existing job offer or to instead continue looking for an even better job, it is not sufficient to only estimate whether better jobs exist and how much better they are than the current offer. Instead, it is also necessary to estimate whether we are capable of getting any of those potentially better jobs and, if we are, whether the investment and persistence that will be necessary are worth the eventual payoff.

Of particular relevance to this latter point is the emerging literature on “grit”, which is defined as the “perseverance or passion for long-term goals” [17]. Individuals who possess high levels of this construct tend to stick with tasks over the long term, even in the face of difficulty [18]. They are also generally more likely to experience greater life success than their less gritty counterparts. For example, individuals high in grit have been shown to have more academic success [19,20], career success [21,22], and overall life satisfaction and happiness [23] as compared to individuals with lower levels of this trait. Grittier individuals are also more likely to successfully complete their training if they are in the military, hold on to their jobs, finish college, and have successful marriages [22] than less gritty individuals.

While it is known that individuals with higher amounts of grit tend to persevere in tasks longer than individuals with lower amounts of grit, as discussed above, the decision to persevere/explore should ideally be related both to the belief that something better than the current status quo exists AND that one is capable of actually achieving that something better. It is unclear, however, which of those inferences is more strongly related to grit. For example, do grittier individuals believe that there are larger rewards available than less gritty individuals, or do gritty and non-gritty individuals both make the same inference regarding what rewards are available but gritty individuals infer that they are more likely to actually achieve those rewards?

To begin to address this question we developed two “exploration” and two “persistence” measures. In the two exploration measures (Chain Task and Grid Task–see below), participants were repeatedly given the opportunity to either exploit a known reward or to explore for new and potentially larger rewards. Critically, these two exploration tasks were designed in such a way that there was no reason for a participant to believe that if a better reward existed, that they would personally be unable to obtain it based on their assessment of their own abilities. Conversely, the two persistence measures (the Impossible Remote Associates Task and the Impossible Raven’s Advanced Progressive Matrices; IRAT and IRAPM respectively) were designed to exclusively tap into participants’ beliefs about their own abilities. In these two tasks, participants were given puzzles to solve, and they could either persist in attempting to find a correct answer, or could quit and move on to a new problem. Unbeknownst to the participants, some of the problems were designed to be impossible to solve to ensure that participants were forced to quit and move on [24,25]. Importantly, and contrary to the Chain and Grid tasks, these two persistence tasks were designed in such a way that they did not give rise to varying beliefs about the presence of reward, as participants were given strong reason to believe that a reward always existed in both tasks (i.e., a correct answer). Dispositional grit was independently assessed using the Grit-S questionnaire [26]. Finally, to independently assess actual cognitive abilities (i.e., abilities upon which performance on the Remote Associates Task and Raven’s Advanced Progressive Matrices depend) working memory performance was measured.

We anticipated two possible outcomes. On the one hand, individuals who are high in grit/persistence might only persist when there is a known reward and the individual has some inclination of their own ability to achieve the reward. If that were the case, we would expect to see a significant relationship between grit and performance on our two persistence tasks, but not between grit and performance on our exploration tasks. On the other hand, greater exploratory behaviors might be associated with, and even require, a certain grittiness or persistence. Individuals who are more likely to persist might also be more willing to try multiple options in the exploration tasks in order to obtain higher rewards, whereas individuals who are less persistent might take the easily obtained, but small, rewards on these tasks. In that case, we would expect a positive relationship between all of the measures. To examine these various issues, we first ran a smaller pilot study (Experiment 1A; N = 30) and then followed up with a larger sample/higher powered study (Experiment 1B; N = 95). The latter experiment also included a measure of the well-established personality factor of conscientiousness in order to assess the extent to which grit predicted variability above and beyond what was accounted for by conscientiousness.

## Experiment 1A: Method

### Participants

A total of 30 University of Wisconsin-Madison undergraduate students (21 female, 9 male) participated in this study. The participants ranged in age from 18 to 22 years (*M* = 19.3, *SD* = 1.12).

### Ethics statement

All subjects provided written consent and course credit for their participation in both Experiment 1A and 1B. This study was approved by the Education and Social/Behavioral Science Institutional Review Board (IRB) at the University of Wisconsin-Madison.

### Stimuli and design

#### Apparatus

All computerized tasks were created and controlled using MATLAB and the Psychophysics Toolbox (PTB-3) [27–29]. Tasks were performed in a dimly-lit testing room on a Dell OptiPlex 780 computer with a 23-inch flat screen monitor. Participants had an unrestrained viewing distance of approximately 60 cm. All responses were made via manual button press on the keyboard, or with the mouse.

#### Overall design

Participants completed five computerized tasks. Two of the tasks (the Chain Task and the Grid Task) were designed to measure exploration/persistence that was related to participants’ beliefs about the presence and/or amount of reward in the environment, and two of the tasks (IRAT and IRAPM) were designed to measure persistence that was related to the participants’ beliefs about their ability to obtain a reward. Participants completed the tasks in the following order: N-back working memory, IRAPM, Chain Task, Grid Task, and the IRAT. Then, after all five computerized tasks were completed, participants completed the 8-item Short Grit Scale (Grit-S; [26]; Grit-S; reported reliability α = .83). The grit survey was always completed at the end as it is the least likely task to be affected by fatigue.

#### Tasks to measure belief about reward in environment

**Fig 1:**
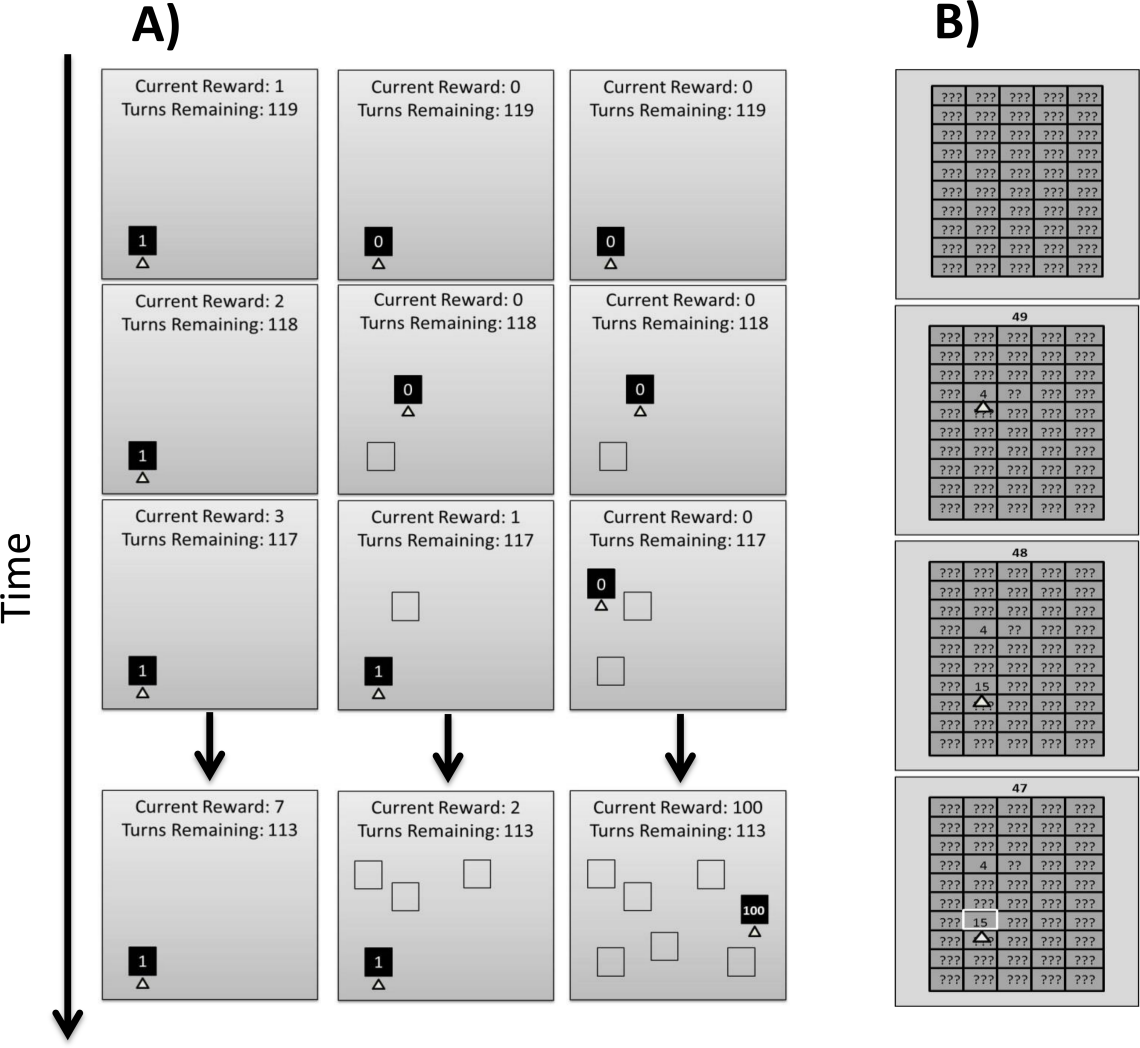
Example of the Chain exploration task and the Grid exploration task. **A)** Example of exploration on the Chain Task. In this task participants press either the “C” or the “I” key in order to earn rewards (points). Pressing the “C” key awards 1 point per turn, with the points always appearing in the same box on the screen (left panel). Pressing the “I” key, on the other hand, results in different boxes being revealed on the screen, all of which contain a 0 point value (center and right panels). On each subsequent press, there is a 20% chance that participants will be returned to the first box and 1 point will be rewarded (center panel). However, there is an 80% chance that a new point box will be revealed after pressing the “I” key, and if this occurs 7 times in a row a 100 point box is revealed (right panel). **B)** Example from the Grid Task. Participants use the mouse to click one of the 50 boxes, and the number at the top of the display indicates how many clicks they have remaining. The participant receives the points revealed in the box, so in this example they received 4 points for their first click (second box from top), and 15 for their second click (3^rd^ box from top). In the bottom box, the participant has decided to not continue clicking boxes as they believe that 15 points is the maximum number hidden in the grid. As such, they can press the right mouse button (indicated by white highlighting) to multiply the points in that box by the number of remaining trials (47 in this example).

**Chain Task (CT).** The overall task structure was identical to versions that have previously been used in the reinforcement learning literature [3,6]. At the beginning of the task, participants were presented with a single yellow box with the number ‘0’ in the center of the box. At the top of the screen was a counter that indicated the number of turns left, and a second counter that indicated the number of points that the participant had been awarded. Participants were told that they could press either the ‘I’ or the ‘C’ key to try to gain rewards, but were not provided with any further information. If participants pressed the ‘C’ key, they would remain in the initial box and the ‘0’ in the box would change to a ‘1’ (i.e., they would be awarded with a single point; see left panel of Fig 1A). If they pressed the ‘I’ key, there was 80% chance of moving to a new box elsewhere on the screen (receiving no points). The remaining 20% of the time the participant was moved to the first box, regardless of where they were at the beginning of the trial, and was awarded one point (see right panel of Fig 1A). While this was unknown to the participants, there were a total of 7 such boxes (or states, as they are referred to in the reinforcement learning literature). Thus, if the participant pressed the ‘I’ key while in the first box, there was an 80% chance of moving to the second box and a 20% chance of staying in the first box. If the participants pressed the ‘I’ key while in the second box, there was an 80% chance of moving to the third box and a 20% chance of moving back to the first box, and so forth until they reached the seventh box. If they pressed the ‘I’ key while in the seventh box, there was an 80% chance of being awarded 100 points and remaining in the seventh box (again, the remaining 20% of the time they would be moved back to the first box and given one point; see center panel Fig 1A). Participants were given 150 turns in total, and were told that the participant who finished with the most points would be awarded a $10 bonus. For those participants who never discovered the large point value (i.e., most participants), their exploration score was calculated as the total number of times that they clicked the ‘I’ key. For those few participants who did discover the large point value, their exploration score was based on the turn number on which they first received the large point value. As such, participants who largely chose to exploit the small reward that came from pressing the ‘C’ key received a low exploration score, and those who persisted in pressing the initially unrewarding ‘I’ key had high exploration scores, particularly if it took a large number of presses in order to uncover the reward.

**Grid Task (GT).** The screen was divided into a 10 x 5 grid of rectangular boxes that were outlined in white on a grey background. Each box initially contained 3 question marks which, when clicked, revealed a point value underneath (see Fig 1B). Point values were randomly assigned to boxes. The values were created by first simulating a normal distribution (*M* = 3, *SD* = 1.25) and then exponentiating the resulting values to produce a log-normal distribution (wherein there were some very high values in the tail of the distribution). Finally, 25% of the boxes were randomly set to 0. Participants were instructed to click boxes in order to earn points. They had the option to click as many different boxes as they liked (up to their total number of clicks for a trial–see below), but were told that if they found a box with a particularly high reward value they could right click the box to have the point value multiplied by the total number of remaining clicks (i.e., mathematically equivalent to simply clicking on that given box for all of the remaining clicks). Participants were given 50 clicks per trial, with 30 trials total, and were told that a $10 bonus would be given to the participant who scored the highest average points throughout the course of the experiment. At the end of each trial, participants were provided with their current average score, as well as their score on the previous trial. The exploration score for this task was calculated by summing the total number of unique box clicks across all trials.

#### Tasks to measure belief about own ability

**Impossible Remote Associates Task (IRAT).** Twelve items were selected from the RAT [30]. For each item, 3 words were presented in black font in the center of a grey background. Participants were asked to guess the word that was common to all three. For example, if they saw “PLATE, LINE, DOG”, the correct answer would be “HOT.” If they thought that they knew the answer, they pressed the left shift key on the keyboard, after which they were prompted to type in their guess. However, if they felt that they could not arrive at the answer, they pressed the right shift key to move on to the next item. The items remained on the screen until the participant made a response, and participants were asked to only enter a guess if they were fairly certain that they knew the answer. For 3 of the 12 items, we replaced one of the 3 words with a word from another triplet in the RAT, thus making them impossible to answer correctly. The impossible items were “MOUNTAIN, BREAD, BIG”, “BONE, TABLE, CURTAIN,” and “STOOL, POWDER, CAR.” During the post-experiment debriefing, all participants reported that they were unaware that there were impossible-to-solve items included in the IRAT. Persistence was measured by calculating the average difference in reaction time (RT) for the impossible items as compared to the answerable items. Actual RAT performance, however, could not be reliably calculated due to the presence of the impossible items, and due to the small number of RAT items that were used.

**Impossible Raven’s Advanced Progressive Matrices (IRAPM).** The IRAPM task utilized, for the most part, the exact same stimuli as are employed in the standard Raven’s Advanced Progressive Matrices [31]. In this task, a pattern is presented with one piece missing on each trial, and participants must select the missing piece from 8 options. In our version, we selected 12 items from the RAPM which gradually increased in difficulty. Participants were asked to select which of 8 possible selections best fit into the missing section for each item, and indicated their choice by pressing the corresponding number key on the keyboard. If they were unable to solve the puzzle, they were instructed to press the right shift key to move on to the next item. Items remained on the screen until the participant made a response, and participants were asked to only enter a guess if they were fairly certain that they knew the answer. There was no time limit for completing the task. Unbeknownst to the participants, 3 of the 12 items (#’s 3, 8, and 10) had the correct choice removed and replaced with a choice from an unused item from the RAPM, thus making it impossible to solve. During the post-experiment debriefing, all participants reported that they were unaware that there were impossible-to-solve items included in the IRAPM. In order to measure persistence, the difference in RT for the first impossible item as compared to the RT for the two surrounding items was calculated. As with the IRAT, typical RAPM performance could not be measured due to possible interference from the impossible items, and due to the limited number of real RAPM items that we presented.

#### Working memory task

The working memory task was a variation on the standard N-back working memory task [32]. The screen space was evenly divided into 7 columns from left to right, and 21 rows from top to bottom. The participant was made aware of this fact during the instructions. The column/row divisions, however, were not explicitly displayed on the screen. Each trial began by indicating the number of columns (between 1 and 7) that were going to be used on that trial. This number corresponded directly to the ‘N’ in the N-back (i.e., on 2-back trials, two columns were used; on 3-back trials, three columns were used, etc.).

For ease of exposition, we will describe the sequence of events for a 2-back trial, and then based upon this describe the other levels of ‘N.’ Participants first pressed a key to begin the trial. After a 1000 ms wait, a single digit (from 1–9) was displayed in the first column and first row position. Then, after 1000 ms the first digit disappeared and a digit was displayed in the second column, first row position. After another 1000 ms, the second digit disappeared and a digit was displayed in the first column, second row position. The participant then needed to determine whether the currently displayed digit was the same as the digit that had appeared directly above it (i.e., that had appeared in the first column, first row position–which was the digit “2-back”). If the digit was the same (50% of the time) the participant pressed the left arrow key, and if it was different the participant pressed the right arrow key. That digit then disappeared and a digit appeared in the next location (second column, second row). Again, the participant needed to indicate whether the current digit was the same as the digit that had been displayed directly above it (second column, first row). The next digit then appeared in the first column, third row, and so forth until the participant had made a total of 20 responses.

The other values of “N” worked in exactly the same fashion as above, but utilizing different numbers of columns. The participants’ task remained the same throughout, such that they were required to indicate whether the currently displayed digit was the same as the digit that had been displayed in the same column, but in the row directly above. Each value of N (from 1–7) was presented four times, for a total of 28 trials. The task always began with N = 1, and then increased by one every two trials up to N = 7. N then decreased by one every two trials until again reaching N = 1. The dependent measure of interest was overall accuracy at each level of N.

## Experiment 1A: Results

### Overall performance

Two participants were removed from the final analysis for obtaining Grid Task exploration scores greater than 3 standard deviations from the mean (i.e., these participants appeared to simply click all the squares, rather than truly maximizing performance), leaving a total of 28 participants. Means, standard deviations, minimum and maximum scores, and skewness and kurtosis values for the 4 exploration measures, overall working memory scores (see below), and the Grit-S, are presented in Table 1.

**Table 1:** Means, standard deviations, ranges, skewness, and kurtosis for all measures.

	*M*	*SD*	Min	Max	Skewness	Kurtosis
Grit-S	3.38	0.54	2.50	4.38	0.20	-0.78
Working Memory	0.83	0.07	0.66	0.96	-0.57	0.65
Chain Task	26.71	24.32	3.00	98.00	1.28	1.29
Grid Task	417.96	102.01	268.00	652.00	0.65	-0.17
IRAT	11.95	13.62	-16.36	37.94	0.19	-0.48
IRAPM	18.27	14.32	-9.99	47.15	0.24	-0.38

For the working memory task, accuracy ranged from 84–100% for the easiest trials (1-back), and from 56% to 94% for the most difficult trials (7-back). There was a significant main effect of difficulty, such that accuracy decreased as the number of columns increased, *F*(6, 162) = 77.27, *p* < .001. In order to examine the relationships between working memory and exploration, an average working memory score was calculated. As most participants performed at ceiling on the 1, 2, and 3-back trials, only accuracy for the 4, 5, 6, and 7-back trials was averaged in order to obtain an index of overall working memory performance. Overall working memory scores are also presented in Table 1.

### Relationships among persistence, exploration, and working memory

As discussed above, the rational decision to explore/persist should be based on two inferences–one related to whether there is a reward to find in the environment, and one related to an individual’s beliefs regarding their ability to obtain rewards that are present. We thus predicted that we would find correlated patterns of individual differences in the tasks that required a common inference (i.e., the Chain Task and the Grid Task = belief about whether there is reward in the environment; the IRAT and the IRAPM = belief about an individual’s ability to obtain a reward), but would not necessarily find any correlations across the two types of inferences. To assess this, we ran independent correlations between all of the four measures (see Table 2). As expected, there was a significant positive correlation between then Chain and Grid Task exploration scores. There was also a significant positive correlation between IRAT and IRAPM persistence scores. However, there were no relationships between Chain Task/Grid Task scores and IRAT/IRAPM scores (all *p*’s > .28).

**Table 2:** Correlations among exploration/persistence, working memory, and grit measures.

	Chain Task	Grid Task	IRAT	IRAPM	WM
Grid Task	.48**	—			
IRAT	-.13	.03	—		
IRAPM	-.21	.08	.64**	—	
WM	-.18	-.13	.65**	.50**	—
Grit	.06	.21	.37	.34	.21

Note

** = *p* < .001.

Next, we assessed the relationship between these exploration/persistence measures and working memory performance. We predicted that, if the behavior in the IRAT and the IRAPM was rationally related to individual’s beliefs about their abilities, persistence on these tasks would be related to working memory performance (i.e., individuals with greater working memory capacity should be more capable of solving the tasks). However, we predicted that there would be no relationship between performance on either the Chain or Grid tasks and working memory performance. To test this, we ran independent correlations between performance on the working memory task and scores on the four exploration/persistence tasks (see Table 2). As expected, there was a strong positive correlation between working memory performance and both IRAT and IRAPM scores, such that individuals who performed better on the working memory task spent more time working on the impossible items in both the IRAT and the IRAPM. Working memory performance, however, was not significantly correlated with scores on either the Chain or Grid tasks.

Finally, we assessed the relationship between our measure of grit and performance on the various tasks. Because the Grit-S is designed to measure persistence, rather than exploration, we predicted that Grit-S scores would be related to scores in the IRAT and the IRAPM, but not the Chain or Grid tasks. To test this, we ran independent correlations between Grit-S scores and the exploration/persistence measures. As expected, while the relationship between Grit-S scores and time on both the IRAT and IRAPM approached significance (*p* = .026 and .038 one-tailed, respectively), there were no relationships between Grit-S scores and Chain/Grid task scores, with correlations of 0.06 and 0.21 respectively.

## Experiment 1A: Discussion

We developed two sets of exploration tasks: one that targeted participant beliefs about the presence/magnitude of rewards, and one that targeted participant beliefs about their ability to achieve a reward. As expected, performance on the “beliefs about rewards” tasks were strongly correlated with one another, such that participants who took a more exploratory approach on the Chain Task were also more exploratory on the Grid Task. Similarly, performance on the “beliefs about ability” tasks were strongly correlated, such participants who persisted on the IRAT task also persisted on the IRAPM task. However, the two types of tasks were uncorrelated. Interestingly, only performance on the “beliefs about ability” tasks (IRAT and IRAPM) was related to an independent measure of grit, although this relationship fell just short of significance. This suggests that individuals who score higher on a measure of grit may be more likely to explore, but only when there is a known reward available that they believe they have the ability to achieve.

We also found support for the hypothesis that working memory would be unrelated to pure exploration behaviors on the Chain Task and Grid Task (as neither task requires significant working memory resources for a participant to be successful), but would be associated with persistence on both the IRAT and IRAPM (as both tasks do require participants to hold several possible solutions in mind simultaneously). Indeed, there was a strong positive relationship between working memory and persistence on both the IRAT and IRAPM tasks, and no relationship between WM and performance on either the Chain Task or Grid Task. This finding suggests that individuals who are more persistent on an impossible task also perform better on a task of working memory, as compared to those who are less persistent (although note that while working memory was numerically positively related with Grit-S scores, this relationship did not reach significance).

Although these results are consistent with our hypotheses, our sample was quite limited in size. As such, we attempted to replicate our findings with a larger sample in a second experiment (Experiment 1b). In addition, a recent meta-analysis has suggested that there may be a strong association between grit and the Big-5 personality trait of conscientiousness [33]. Specifically, Credé et al. [32] showed that scores on the grit measure are strongly correlated with conscientiousness scores and are weakly associated with some measures of performance. However, they also showed that scores on the perseverance facet of the Grit-S scores explained significant unique variance in academic performance over and above conscientiousness, suggesting that while these concepts are highly related, there is a unique aspect of grit that drives behavior. Resolving the debate over whether or not grit is a unique trait is beyond the scope of the current paper, but we nonetheless decided to include a short measure of conscientiousness in Experiment 1b in order to examine whether grit can account for individual differences in persistence/exploratory behaviors over and above conscientiousness.

## Experiment 1B: Method

### Participants

A total of 95 University of Wisconsin-Madison undergraduate students (57 female, 38 male) participated in this study. The participants ranged in age from 18 to 21 years (*M* = 18.8, *SD* = 0.929). All subjects provided written consent and were awarded course credit for their participation. This study was approved by the Education and Social/Behavioral Science Institutional Review Board (IRB) at the University of Wisconsin-Madison.

### Stimuli and design

#### Apparatus

The apparatus was largely identical to that described in Experiment 1A with the exception that tasks were performed in one of five dimly-lit testing rooms using a Dell OptiPlex 780 computer with either a 21.5, 23, or 24-inch flat screen monitor.

#### Overall design

The overall design was also largely identical to that described in Experiment 1A with three key differences. First, as discussed above, participants completed a 10-item Conscientiousness Scale at the end of the experiment [34] in addition to the 8-item Short Grit Scale. Second, because the full battery of tasks tended to extend beyond the allotted time in Experiment 1A, the number of trials in the N-back task was reduced such that each value of N (from 1–7) was presented twice rather than four times. Third, the order in which participants completed the five main tasks (N-back working memory, IRAPM, Chain Task, Grid Task, and IRAT) was chosen randomly (without replacement) from the full set of 120 possible sequences. As in Experiment 1A, the questionnaires (Conscientiousness and Grit) were always completed at the end as they are the least likely to be affected by fatigue.

## Experiment 1B: Results

### Overall performance

Two participants were removed from the final analysis for obtaining IRAPM (one participant) or IRAT (both participants) exploration scores that were greater than 3 standard deviations from the mean. As such, a total of 93 participants were included in the final analysis. Means, standard deviations, minimum/maximum scores, and skewness and kurtosis statistics for all measures are presented in Table 3. For the working memory task, accuracy ranged from 68–100% for the easiest trials (1-back), and from 43 to 98% for the most difficult trials (7-back). There was a significant main effect of difficulty, such that accuracy decreased as the number of columns increased, *F*(6, 552) = 126.83, *p* < .001, η^2^_ρ_ = .58. An average working memory score (4 through 7-back) was calculated in order to obtain an index of overall working memory performance. Overall working memory scores are also presented in Table 3.

**Table 3:** Means, standard deviations, ranges, skewness, and kurtosis for all measures.

	*M*	*SD*	Min	Max	Skewness	Kurtosis
Grit-S	3.48	0.69	2.00	4.88	-0.28	-0.72
Conscientiousness	3.65	0.72	1.80	4.90	-0.50	-0.27
Working Memory	0.81	0.09	0.59	0.99	0.95	0.13
Chain Task	25.53	32.44	1.00	129.00	1.69	2.04
Grid Task	459.08	199.40	95.00	929.00	0.28	-0.53
IRAT	7.47	11.86	-21.89	49.31	0.95	1.40
IRAPM	17.88	19.18	-13.63	83.63	1.03	1.08

### Relationships among persistence, exploration, and working memory

As in Experiment 1A, we predicted that we would find correlated patterns of individual differences in the tasks that required a common inference (i.e., the Chain Task and the Grid Task = belief about whether there is reward in the environment; the IRAT and the IRAPM = belief about an individual’s ability to obtain a reward), but would not find any relationships across the two types of inferences. To assess this hypothesis, we ran independent correlations between all of the four measures (see Table 4). As expected, there was a significant positive correlation between the Chain Task and the Grid Task exploration scores, as well as a significant positive correlation between the IRAT and IRAPM persistence scores. However, there were no relationships between Chain Task/Grid Task scores and IRAT/IRAPM scores (all *p*’s > .49).

**Table 4:** Correlations among exploration/persistence, working memory, grit, and conscientiousness measures.

	Chain Task	Grid Task	IRAT	IRAPM	WM	Grit
Grid Task	.64**	—				
IRAT	-.01	.07	—			
IRAPM	.05	.01	.58**	—		
WM	-.15	-.02	.36**	.48**	—	
Grit	-.01	-.06	.18	.29**	.09	—
Conscientiousness	-.05	-.02	.22*	.46**	.24*	.58**

Note

* = *p* < .05.

** = *p* < .001.

Next, we assessed the relationship between the exploration/persistence measures and working memory performance. Given the results of Experiment 1A, we expected that persistence on the IRAT and IRAPM would be related to working memory performance. We also predicted that there would be no relationship between performance on either the Chain or Grid tasks and working memory performance. To test this, we ran independent correlations between performance on the working memory task and scores on the four exploration/persistence tasks (see Table 4). As expected, there was a moderate positive correlation between working memory performance and both IRAT and IRAPM scores, such that individuals who performed better on the working memory task spent more time working on the impossible items in both the IRAT and the IRAPM. However, there were no relationships between working memory scores and scores on the Chain/Grid tasks.

We then assessed the relationships between our measures of Grit and conscientiousness, and performance on the various tasks. We anticipated that, as in Experiment 1A, Grit would be related to scores in the IRAT and the IRAPM, but not the Chain or Grid tasks. Additionally, we predicted that conscientiousness would likewise be associated with IRAT and IRAPM scores, but not with scores on the Chain and Grid tasks. To test this, we ran independent correlations between Grit-S scores and the exploration/persistence measures (see Table 4). As expected, there were no relationships between Grit-S/conscientiousness scores and scores on the Chain/Grid tasks (all p’s > .50). Furthermore, both Grit-S scores and conscientiousness scores were significantly positively correlated with IRAPM persistence time. However, while conscientiousness scores were also positively associated with scores on the IRAT, the relationship between Grit-S and IRAT persistence time was small and failed to reach significance (*p* = .08).

Finally, a stepwise multiple regression analysis was conducted to examine whether working memory, Grit-S, and conscientiousness scores were significant predictors of scores on the IRAPM. We were particularly interested in examining whether Grit and conscientiousness explained unique variance in IRAPM scores, as these measures were not only significantly associated with the IRAPM, but were also strongly associated with one another. In this analysis, only working memory (*sr*^*2*^ = .149, *p* < .001) and conscientiousness (*sr*^*2*^ = .123, *p* < .001) were significant unique predictors of IRAPM scores, and together they explained a significant 35.6% of the variance in IRAPM persistence scores, *R* = .597, *F*(2, 90) = 24.92, *p* < .001.

### Additional exploratory factor analysis

In order to provide converging evidence for the patterns observed in the simpler correlational analyses, we performed an exploratory factor analysis with varimax rotation that included 6 of our measures (i.e., the 4 exploration measures, Grit-S, conscientiousness). We obtained a 3-factor solution, with the Grid and Chain exploration tasks loading onto one factor, the IRAPM and IRAT onto another factor, and the Grit-S and Conscientiousness measures loading onto a third factor (see Table 5 for factor loadings). We then examined the correlation between WM and each of these 3 factors. WM was significantly correlated with the IRAPM/IRAT factor (*r* = .513), but was not significantly related to the other two factors.

**Table 5:** Rotated factor loadings for experiment 1b showing three distinct factors.

	Component		
	1	2	3
IRAPM	.869	.004	.275
IRAT	.915	.087	.072
Chain Exploration	.034	.904	.019
Grid Exploration	.052	.900	-.038
Grit	.096	.004	.891
Conscientiousness	.225	-.025	.859

*Method*: Principal Component Analysis. Varimax with Kaiser normalization.

## Experiment 1B: Discussion

The findings in Experiment 1B largely replicated Experiment 1A. As expected, participants who took a more exploratory approach on the Chain Task were also more exploratory on the Grid Task, and participants who persisted on the IRAT task also persisted on the IRAPM task, but there was no relationship between the two different categories of task. In addition, Grit-S scores were related to the performance on the “belief of ability” measures (although this did not reach significance in the case of the IRAT task), but not the “belief of rewards” tasks. Finally, there was an association between WM scores and performance on the IRAT and IRAPM, but no relationship between WM and scores on either the Grid or the Chain task.

Given that recent research has indicated that grit is strongly related to conscientiousness (and indeed, it has been suggested that Grit can be fully or near fully subsumed under the traditional construct of conscientiousness; [33]), we also examined the relationships among grit, conscientiousness and exploration. As expected from previous work, Grit-S and conscientiousness scores were highly correlated (indeed, this relationship is the strongest of all of the pairwise correlations observed in the current study). Perhaps not surprisingly then, conscientiousness, like Grit-S, was also correlated with persistence on the “belief of ability” tasks (IRAPM and IRAT), but not with scores on the Grid or Chain tasks. Conscientiousness scores were also weakly correlated with WM scores. Critically, when Grit-S, conscientiousness, and WM scores were entered into a multiple regression analysis, only conscientiousness and working memory ability were found to explain significant unique variance in IRAPM persistence time (i.e., once the variance predicted by conscientiousness was accounted for, Grit-S had no predictive value). This suggests that it may not be grit per se that explains exploration behaviors when the reward structure is known, but rather some underlying construct shared by both grit and conscientiousness.

## General discussion

Previous research has demonstrated that individuals who score highly on measures of grit, and who persevere in the face of difficult tasks, are often more successful in a variety of domains including health, education, and finances [20,22,23,35,36]. It remained unclear whether grittier individuals persist because they believe there are more likely/larger rewards available in the environment than do individuals with less grit, or if instead gritty and non-gritty individuals both make similar inferences regarding what rewards are available in the environment, but gritty individuals infer that they are more likely to actually reach those rewards. Here we developed two pairs of tasks: one designed to measure the former inference (i.e., where persistence is largely dependent on whether one believes that large rewards are available), and one designed to measure the latter inference (i.e., where persistence is largely dependent on whether one believes that they are personally capable of obtaining the reward). As expected, performance on these pairs of tasks was strongly correlated, such that participants who took a more exploratory approach on the Chain Task were also more exploratory on the Grid Task, and participants who persisted on the IRAT task also persisted on the IRAPM task. Interestingly, however, only performance on the persistence tasks (IRAT and IRAPM) was related to an independent, and well-established, measure of grit. This suggests that individuals higher in perseverance or grit are not necessarily more willing to explore given uncertain rewards, but instead are only more likely to continue when there is a known reward available.

In a second experiment, we replicated the findings of the first with a much larger dataset and demonstrated that while grit was still associated with performance on the IRAPM, and to a lesser extent the IRAT (albeit nonsignificant), these relationships were no longer significant once working memory and conscientiousness had been accounted for. This suggests that it is not necessarily “grit” per se that leads individuals to spend more time on impossible tasks, but rather some underlying construct shared by both conscientiousness and grit, such as persistence or determination, that leads to these behaviors. Regardless of the measure used, though, the finding that grit/conscientiousness was associated with longer exploration time, but only on the IRAT and IRAPM, was consistent across both experiments.

One explanation for the finding that grit/conscientiousness was only associated with our “beliefs about ability” tasks, as opposed to our “beliefs about rewards” tasks, could be that for the IRAT and IRAPM, participants had some prior knowledge of their ability to tackle the kinds of problems presented in these tasks. Indeed, previous research has shown a strong link between grit/persistence and metacognition, such that individuals higher in grit are more likely to have knowledge and better regulation of their own strategies during problem solving tasks [37]. Similarly, there is a strong positive correlation between conscientiousness and metacognition [38], as well as between conscientiousness and persistence on a boring task [39]. As such, it follows that these grittier/more conscientious individuals had a better sense for their own limits when it came to solving problems on the IRAT and IRAPM, and were thus less likely to give up knowing that, based on past experiences, they are typically able to succeed on these types of tasks. This is also in line with our finding that working memory performance was associated with IRAT and IRAPM exploration, suggesting that those who felt more equipped to succeed on the task were more likely to persist.

In addition to the expected relationships, we did find a marginal inverse relationship between exploration on the Chain Task and persistence on the IRAPM (and a smaller inverse relationship between the Chain Task and IRAT) in Experiment 1A. This raised the interesting possibility that reward-seeking in the face of ambiguity, and the tendency to try riskier options (such as in the Chain Task) in order to receive larger rewards, is not associated with persistent tendencies, and could in fact be associated with *less* persistence (it should be noted, however, that this inverse trend was not apparent with the Grit-S scores). In other words, individuals who base their decisions more strongly upon their own abilities might prefer to avoid pure exploration and vice versa. However, in Experiment 1B we essentially found no relationship between grit/conscientiousness and performance on either the Grid or the Chain task, and virtually no relationship between the two types of exploration tasks. Researchers should nonetheless consider the possibility that more persistent individuals might be less willing to try high-risk, high-reward options, particularly when they have no way to gauge their ability to succeed on a task. It could be that more persistent individuals are successful, but not necessarily high achievers given that they may switch to an exploitation strategy early on. It would thus be interesting to examine how persistent individuals perform on reward tasks with varying levels of uncertainty and reward magnitude.

The second goal of this study was to examine the role of working memory capacity in performance on our four tasks. We anticipated that working memory would be related to persistence on the IRAT and IRAPM tasks given that both tasks require the use of prior knowledge or the ability to simultaneously weigh several options. Thus, if individuals are aware of their own cognitive abilities and use this information to determine whether or not to persist, those with higher working memory scores should tend to persist longer than those with lower working memory scores. On the other hand, working memory should be unrelated to pure exploration behaviors on the Chain Task and Grid Task (as neither requires any particular individual cognitive abilities in order to be successful). We found support for this hypothesis as there was a strong positive relationship between working memory and persistence on both the IRAT and IRAPM tasks across both experiments, and no relationship between WM and performance on either the Chain Task or Grid Task. This finding may suggest that individuals who are more persistent are also better on tasks of working memory than those who are less persistent (although note that while working memory was numerically positively related with Grit-S scores, this did not reach significance).

The strong patterns of correlated individual differences suggest two possible and potentially complementary future routes for manipulating behavior. One route would be by manipulating participants’ beliefs about the presence or absence of reward in the environment. Stronger beliefs that rewards exist should in turn promote increased exploration and persistence. The second route would be by manipulating participants’ beliefs about their own ability to obtain rewards in the environment. Again, stronger beliefs that one is capable of obtaining whatever rewards are found should also in turn promote increased exploration and persistence.

Although it comes at the problem from a slightly different perspective, these results are also fully consistent with a well-described and elaborated framework in the domain of motivational psychology known as the Expectancy-Value Theory of Motivation [40–43]. This framework posits that the motivation to engage in an action (e.g., to make achievement-related choices in formal schooling), springs from a number of factors. Amongst these factors are 1) whether the individual values successfully performing the task; 2) whether they believe they have the ability to perform the task; and 3) whether they believe they can improve their performance on the task. Although they are clearly not identical, the first factor (whether they value successfully performing the task) in Expectancy-Value theory has clear similarities to what we have described as the “belief about rewards”. Both, in essence, capture whether exploration is likely to lead to something the individual finds worthwhile. Similarly, there are analogies between what we have labeled as “belief about abilities” and the latter two factors in Expectancy-Value theory (belief about current abilities on the task/ability to improve on the task).

All three of these factors suggest that with additional investment of energy, there is likely to be a concomitant increase in the probability of reward. One consistent question in this domain is the extent to which beliefs about ability are accurately rooted in knowledge about self (i.e., if an individual has the internal belief that they are not capable of performing a task well or learning to perform a task, are they accurate in that assessment?), with some prominent theories suggesting that many individuals underestimate their internal abilities. These theories then suggest that manipulations that increase one’s belief in ability (e.g., Mind Set–[40–43]) will result in actual changes in achievement. While our results do not address that particular issue, to the extent that WM at least partially indexes cognitive abilities that are related to solving problems in the RAT and RAPM, the correlations between WM and IRAPM/IRAT suggest that such internal estimates may be (at least partially) rooted in accurate estimates of self.

Finally, it is worth noting that the methodology, particularly in the case of the IRAPM and the IRAT, can likely be improved upon in future work. In both tasks, a number of the “possible” problems were, in practice, difficult enough that they were, from the perspective of many of the participants, “impossible”. For instance, even at a reasonably academically competitive school such as the University of Wisconsin-Madison, the problems in the RAPM quickly become sufficiently difficult that a reasonable portion of the undergraduates begin to struggle or fail to come to the correct solution. In addition, in the case of RAT, some of the problem sets required knowledge of idiomatic expressions that may not be familiar to the current generation (e.g., “Monster Mash”–a novelty song frequently played in and around Halloween). Because our measures of persistence involved calculating how much longer participants spent on the “impossible” problems as compared to what was expected given their time to complete the “possible” problems, the inclusion of “possible” problems that were, in practice, impossible for the participants would necessarily reduce the quality of the measure. As such, in future studies it would likely be worthwhile to use a slightly easier set of “possible” problems (e.g., the Raven’s Standard Progressive Matrices).

To summarize, over the course of two experiments we demonstrated that the decision to continue to explore, or to cease exploration and move on/settle, can be predicted by individual differences in grit/conscientiousness, such that individuals higher in grit/conscientiousness were more likely to persist on an impossible task. Critically, this relationship was only apparent for those tasks where there was a known reward available that participants believed they had the ability to achieve. Going forward, more research is necessary in order to understand the precise mechanisms of conscientiousness that lead to a greater tendency to explore, as well as why this relationship between grit/conscientiousness was only apparent on a subset of tasks (i.e., “the beliefs about ability” tasks). We highlighted a few suggestions for how future research could proceed, such as by manipulating beliefs about both the possibility of rewards of varying magnitudes in the environment, and beliefs about one’s own ability to achieve a reward, in order to better understand the circumstances under which an individual decides to explore/exploit. Given how critical exploration/exploitation behaviors are to our everyday functioning, and to general success in life, it is worthwhile to better understand the factors that give rise to the decision to persist or settle.
